# Prospect of Induced Pluripotent Stem Cell Genetic Repair to Cure Genetic Diseases

**DOI:** 10.1155/2012/498197

**Published:** 2012-02-12

**Authors:** Jeanne Adiwinata Pawitan

**Affiliations:** Department of Histology, Faculty of Medicine, Universitas Indonesia, Jakarta 10430, Indonesia

## Abstract

In genetic diseases, where the cells are already damaged, the damaged cells can be replaced by new normal cells, which can be differentiated from iPSC. To avoid immune rejection, iPSC from the patient's own cell can be developed. However, iPSC from the patients's cell harbors the same genetic aberration. Therefore, before differentiating the iPSCs into required cells, genetic repair should be done. This review discusses the various technologies to repair the genetic aberration in patient-derived iPSC, or to prevent the genetic aberration to cause further damage in the iPSC-derived cells, such as Zn finger and TALE nuclease genetic editing, RNA interference technology, exon skipping, and gene transfer method. In addition, the challenges in using the iPSC and the strategies to manage the hurdles are addressed.

## 1. Introduction

Since the first generation of induced pluripotent stem cell (iPSC) from mouse adult fibroblast using four inducing factors by Takahashi and Yamanaka in 2006 [1], followed by the generation of human iPSC [2], various vectors to introduce various inducing factors have been published, and various combinations of the inducing factors, in the form of transcription factors or microRNA, were used. Further, there are chemical compounds, for example, butyrate that may enhance the inducing capacity of the transcription factors [3], so that Oct 4 alone is enough to induce somatic cells into iPSC [4].

Further, various patient-derived iPSCs were developed that may be used to reveal the pathogenesis of various genetic diseases. These genetic abnormality-harboring iPSCs may be repaired, and the genetically repaired iPSC may be differentiated into normal required cells [5]. In the future, these patient-derived normal cells may be used to a patient-tailored therapy to replace the damaged cells due to the disease.

To date, iPSCs for various genetic diseases have been developed, such as for certain type of Parkinson's disease [5], spinal muscular atrophy [6], lentigines, electrocardiographic abnormalities, ocular hypertelorism, pulmonary valve stenosis, abnormal genitalia, retardation of growth, and deafness (LEOPARD) syndrome [7], long Q-T syndrome [8], Timothy syndrome [9], Hurler syndrome [10], epidermolysis bullosa [11], and thalassemia [12].

The iPSC resembles embryonic stem cell in the differentiation capacity into various kinds of cells and in inducing teratoma in laboratory animal [1]. However, various researches have shown that iPSC is not identical to embryonic stem cell. Moreover, various aberrations, which may arise during induction or subsequent propagation, pose challenges in the use of iPSC for the cure of genetic diseases.

Therefore, this review discusses the prospect of iPSCs to cure genetic disease, in term of the efficient methods for genetic repair that may be used to repair genetic disease-harboring iPSCs, and the challenges that should be resolved when iPSCs are to be used to cure genetic diseases.

## 2. Methods for Genetic Repair

To date, there are several efficient methods for genetic repair of genetic diseases, that is, zinc finger and transcription activator-like effector (TALE) nuclease method, RNA interference (RNAi), exon skipping technology, and gene transfer. However, when the cells are already damaged, they should be replaced by new normal cells, which can be differentiated from iPSC. Those methods may be used to repair the genetic disease-harboring cells that may be done either in the somatic cells before induction to pluripotency [13], or somatic cell derived iPSC [5].

### 2.1. Zinc Finger Nuclease Method

**Figure 1:**
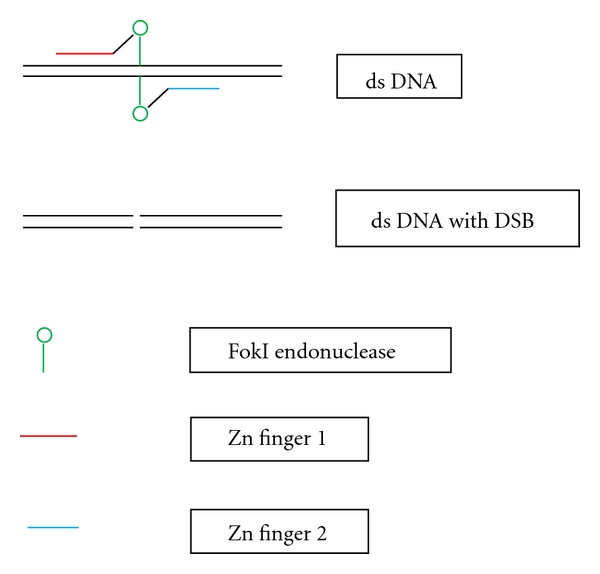
Generation of a double strand break by zinc finger nucleases, ds: double strand, DSB: double strand break, Zn: zinc.

**Figure 2:**
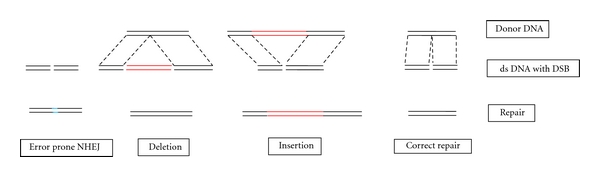
Possibilities of genetic repair using zinc finger nucleases, ds: double-strand, DSB: double-strand break, NHEJ: non-homologous end joining.

The zinc finger nuclease method is one of the efficient genetic editing methods. A Zn finger nuclease consists of a Zn finger domain and FokI endonuclease. The Zn finger domain contains Zn finger motifs that recognize and bind to a specific DNA sequence. The FokI endonuclease works as a dimer to cause a double-strand break (DSB) in the DNA. Therefore, Zn finger nucleases should work in pairs. One of the Zn finger motifs recognizes and binds to the sequence up stream and the other to the sequence down stream to the site to be cleaved by the endonuclease (Figure 1). Principally, a certain Zn finger nuclease can be engineered to recognize any specific sequence and to cause a DSB at any specific site. The DSB is then repaired by homologous recombination, which is facilitated by the presence of exogenous donor DNA homologous to the sequence to be repaired, or by error-prone nonhomologous end joining [14, 15]. To deliver the Zn finger nucleases into a cell, an expression vector containing the Zn finger nucleases can be engineered. The results of this genetic editing may be either mutation repair or insertion of a certain DNA sequence, when a certain exogenous donor DNA is used, or error prone repair when no donor DNA is used, or deletion when two pairs of Zn finger nucleases are used and causing 2 DSB [15]. Therefore, this method may be used to correct a mutation, or to insert or delete a certain DNA sequence (Figure 2).

A study used exogenous donor DNA that was packed in a double-stranded plasmid, or in the form of a single-strand oligodeoxynucleotide. This method was successfully used to repair a point mutation A53T (G209) in *α*-synuclein gene in a Parkinson's disease patient-derived iPSC. Further, the repaired iPSC was successfully differentiated into functional dopaminergic neurons [5].

A drawback of this method is off target DSB due to homodimerization, which may cause undesired mutation or cytotoxicity. Therefore, genome-wide putative-off target mutation assay should be performed, to ascertain that there is no off-target mutation in the genetically repaired iPSC [5]. Recently, to reduce off-target DSB, engineered FokI nuclease that cannot form a homodimer was developed. This method showed that the obligate FokI heterodimer greatly reduces the off target DSB [16, 17].

### 2.2. TALE Nuclease Method

Transcription activator-like effectors from a plant pathogen, the *Xanthomonas sp.*, are sequence-specific DNA-binding proteins. As Zn fingers, TALEs can be engineered to bind to any specific sequence, and linked to a FokI nuclease to work in pair and cleave the sequence [18, 19]. This method was tested in iPS and showed that TALE nuclease mediated site-specific genetic modification with similar precision and efficiency as Zn finger nuclease [20], but with lower degree of off target activity and cytotoxicity [21].

### 2.3. RNA Interference (RNAi) Technology

RNA interference involves micro- (mi-)RNA and small interfering (si)RNA, which, upon base-pairing to their target sequence in a certain mRNA, cause degradation or prevent translation of the mRNA [22]. This method may be useful to suppress the expression of a toxic mutant allele that causes the symptoms of a certain genetic disease. However, this method does not repair the underlying genetic aberration. Therefore, to suppress the expression of the mutant allele in a genetically abnormal iPSC, a method to continuously deliver the interfering RNA is needed.

Various expression systems for either miRNA or siRNA have been developed using various vectors and promoters [23–27]. The expression system for siRNA involves the formation of short hairpin (sh)RNA before the formation of a double-strand functional siRNA, while that for miRNA involves the formation of primary miRNA transcripts, followed by the formation of pre-miRNA, and finally a functional mature miRNA [23, 24].

However, the use of strong promoter results in high level expression of miRNA or shRNA that may lead to cytotoxicity [28–30]. Cytotoxicity of siRNA expression system may be due to competition of the artificial with the natural RNA interference system and lead to disruption of the natural system [31], or off-target silencing [32], possibly due to miRNA-like binding of siRNA at the 3′ UTR region [33]. In addition, shRNA or the viral vector may induce cellular interferon response that leads to universal silencing [34, 35].

Comparison between miRNA and siRNA expression system showed that siRNA was more potent [36], but miRNA expression system was safer [31, 37, 38]. Cytotoxicity and off target effect of siRNA can be reduced by reducing the siRNA concentration [33]. Therefore, using less potent promoter in siRNA expression system may resolve the problem. Alternatively, engineering a single nucleotide bulge in the siRNA expression system may overcome the problem [39].

This RNA interference technology was proven useful to suppress the expression of a mutant allele in Alzheimer's disease in cell culture [40], spinocerebellar ataxia [23], Huntington's disease [41], and amyotrophic lateral sclerosis, in animal models [42, 43].

### 2.4. Exon Skipping Technology

Exon skipping technology causes deletion of selected exon(s) by targeting a sequence in the adjacent intron using an antisense oligonucleotide. This method can be used in genetic aberration where there is a mutation that causes a frameshift or a stop in the mRNA, and deletion of one/several frame-shifted exon(s) leads to a shorter, but still functional protein, for example, dystrophin in Duchene muscular dystrophy (DMD) [44].

However, the use of exogenous antisense oligonucleotide to cause exon skipping in iPSC needs continuous supply of the antisense oligonucleotide. Therefore, to repair a genetic aberration in iPSC, an expression vector needs to be engineered.

A study on DMD mouse model used an expression vector to deliver the antisense oligonucleotide by a single-dose injection into skeletal muscle. The expression vector was engineered using AAV-2-based vector combined to a modified U7 small nuclear (sn)RNA, which was linked to the antisense sequence to both flanking intron of the exon(s) to be deleted. The U7 snRNA guides the antisense sequence to the proper subcellular site and facilitates splicing and exon skipping. The study showed a sustained production of functional dystrophin and correction of the muscular dystrophy [44]. Another study used the same method but administered the expression vector into the hippocampus and showed normalized synaptic plasticity [45].

### 2.5. Gene Transfer Method

Gene transfer method may be useful in genetic diseases where there is genetic aberration that causes the absence of expression of a certain gene, such as in tyrosinemia type 1 due to fumarylacetoacetate hydrolase (FAH) deficiency [46], *β* thalassemia [12], and Fanconi anemia [13]. A study has developed an iPSC from an FAH deficient mouse, corrected the genetic aberration by transduction of FAH cDNA using lentiviral vector, and successfully generated healthy mice from the corrected iPSC [46].

## 3. Challenges in Using iPSCs

As iPSC resembles embryonic stem cell in teratoma-inducing capacity, and the detection of various genetic and epigenetic aberrations, caution should be paid to solve these problems. Moreover, delivery route and the high cost in this patient-tailored therapy may pose other problems. Further, there is still a question of which cell type should be differentiated and transplanted, whether the differentiated iPSCs can integrate and cooperate with other cells in the target site, and whether the differentiated iPSC is not rejected by the immune system. Finally, safety issues concerning the use of genetically repaired iPSC need to be considered.

### 3.1. Teratoma-Inducing Capacity

Theoretically, iPSC can be differentiated into any required cells. A study on human embryonic stem cells used multiple passages under differentiation-inducing condition to eliminate residual tumor-forming cells and proved the absence of pluripotent cells using Oct3/4 marker, and by grafting the differentiated cells in rats [47]. However, human iPSC line-derived dopaminergic neuron progenitor transplantation in a rat model showed the presence of Nestin-positive tumor-like cells at the site of transplantation [48].

Therefore, to prevent tumor formation in patients receiving transplantation of differentiated iPSC, it should be ascertained that the differentiated cells are free from residual pluripotent iPSC, and methods should be developed to purify the differentiated iPSC and to check the absence of tumorigenic potential in the desired cells.

### 3.2. Genetic and Epigenetic Aberrations

A recent study showed that there were variations in the copy number of certain genes in the form of duplications and deletions, in human iPSC [5, 49–51]. Another study showed that there were point mutations in certain somatic protein-coding genes [52].

Apart from teratoma-inducing capacity of iPSC, copy number variation may be present in the form of amplification of oncogenes or deletion of tumor suppressor genes that may lead to tumor formation. Moreover, point mutation may lead to either up regulation or down-regulation of certain important genes other than tumor-related genes. Therefore, caution is warranted before iPSC-derived cells are used in therapy. Substantial genetic abnormalities may be observed by chromosomal analysis, but subtle changes need more careful examination.

An epigenetic study showed aberrant DNA methylation of certain single bases in human iPSC [53]. Moreover, comparison of human iPSC developed from various types of cells representative of ectoderm, mesoderm, and endoderm revealed retention of transcription memory of the original cells due to incomplete promoter DNA methylation [54]. A study on DNA methylation and transcription profiles of 20 different human embryonic stem cell and 12 iPSC lines showed large variations [55]. Analysis of methylation and transcription profiles [54], and transcription and expression profiles enable the prediction of a cell line efficiency to be differentiated into a certain required cell type [56].

Therefore, before attempts to induce iPSC from a patient, determination of the iPSC-derived cells that are required to replace damaged cells may be useful in choosing the cell source for iPSC induction. Using a cell from the same germ layer as the desired differentiated cell is an advantage.

### 3.3. Delivery Route

Delivery route depends on the cells, tissue, or organ to be repaired, which may not be the same for all diseases. For some diseases, the target site may be difficult to reach, for example, Alzheimer, Parkinson's, and other neurological disease, where the target site is inside the brain. Attempts to deliver cells into the brain have been done and showed variable results [57, 58]. Therefore, the simplest way is intravenous delivery. However, it is still a question whether the cells home to the intended target site.

A study on intravenous injection of either bone marrow mesenchymal stem cells or epidermal neural crest stem cells in an animal model showed that both types of cells homed into inflamed corpus callosum. The result of the study suggests that intravenous delivery may work on neurodegenerative diseases where inflammation is present on the target site [59].

Therefore, for each genetic disease, studies are highly needed to identify the most effective, efficient, and safe route of delivery.

### 3.4. High Cost in Developing a Patient-Tailored Therapy

The possibility to repair genetic defect in patient-derived iPSC may lead to individual patient-tailored therapy for genetic diseases. This approach has an advantage compared to allogenic cell therapy, as no immunosuppressive regiment is required, though a study showed that autologous iPSC may elicit immune response [60]. However, the presence of genetic abnormality in various iPSCs necessitates careful screening to ensure the safety of iPSC-derived differentiated cells. To date, technologies to check subtle abnormalities, such as copy number variation and genome wide mutation analysis, are available, though they need a high cost. Therefore, for each genetic disease, development of efficient, effective, and economical method to screen and check the safety of the desired cells is highly needed.

### 3.5. Type of Cell to Be Transplanted and Integration into the Target Site

Requirement of cell type depends on the damaged cells due to the genetic disease. The option is whether to use fully or partly differentiated iPSC. Transplantation of partly differentiated iPSC is intended to resume the differentiation *in vivo* into the mature desired cells.

A study showed that *in vitro* differentiated murine iPSC-derived neurons functionally integrated into the brain and alleviated the symptoms in Parkinson's rat model [61]. Another study on embryonic stem-cell-derived neural stem cells that were transplanted in the putamen of a Parkinson's disease animal model showed that the transplanted cells differentiated *in vivo* into functional dopaminergic neurons [62].

Therefore, studies are required for each genetic disease to determine which cell type and degree of differentiation give the best result in term of cell function, integration, and cooperation with surrounding cells, which finally alleviate the symptoms.

### 3.6. Immune Rejection Problem

Using patient's own cells to provide iPSC-derived cells is believed to handle immune rejection problems. However, a recent study showed that even the patient's own iPSC may induce immune rejection [60]. Moreover, when viral vectors are used to engineer the expression vector for the various methods for genetic repairs or to repress the symptoms, immune rejection may be developed towards the viral vectors. A study showed that viral vectors induced adaptive immune response *in vivo* [63], which leads to inflammatory responses [64]. In addition, innate immune rejection may be developed towards the RNA in case exon skipping or RNA interference method is used. Several studies showed interferon production due to RNA-expressing vectors *in vitro* [34, 35], which can be overcome by reducing the length of RNA to below 21-mers [35].

Although in iPSC genetic repair, the expression vector is transduced into the iPSC, and the presence in blood or tissue may be minimal to be able to induce immune response, studies are highly needed to find a method to cope with immune rejection problems and to ascertain that the corrected iPSC is really safe and will not be rejected.

### 3.7. Safety Issues in Using Genetically Repaired iPSC

Most of the methods of genetic repair, which may be used to repair patient-derived iPSC, use viral vectors as expression vectors, such as lentiviral-based vector in gene transfer technology [13], TALE nuclease genetic editing [18], RNAi technology [24, 25], or AAV-based vector in RNAi technology [29] and exon skipping technology [44, 45].

Viral vectors especially lentiviral-based vectors are known to cause side effects that range from immortalization to clonal dominance *in vitro*, and oncogenesis *in vivo*, due to integration of the vector into host genome. The integration or insertional mutagenesis activates the expression of a protooncogene or cancer promoting genes near the integration site [65].

Therefore, it is very important to address the clinical safety of the vectors. This purpose can be achieved by deletion of promoter element in the viral long terminal repeat (LTR), which is termed self-inactivating (SIN) LTR, which may significantly decrease cellular transformation *in vitro*. Another approach to reduce oncogenesis is by insertion of an insulator element into the LTR [65]. Thus, vector design and safety assessment of the vector before constructing an expression vector for the purpose of genetically repairing iPSC is of high importance.

## 4. Conclusion

Induced pluripotent stem cells are very promising as the source of the required cells to replace the damaged cells in various genetic diseases. However, further studies are needed to resolve the various challenges.
